# P-332. A Discrete Choice Experiment to Evaluate Healthcare Personnel Preferences Regarding Risk-Tailored Policies for Contact Precautions for Patients with Methicillin-Resistant *Staphylococcus aureus* in Hospitals

**DOI:** 10.1093/ofid/ofae631.535

**Published:** 2025-01-29

**Authors:** Lyndsay M O’Hara, David P Calfee, Nicholas Angelino, Anthony Harris, Graham M Snyder, Elise Martin, Nathan N O’Hara

**Affiliations:** University of Maryland School of Medicine, Baltimore, Maryland; Weill Cornell Medicine, New York, New York; University of Maryland School of Medicine, Baltimore, Maryland; University of Maryland School of Medicine, Baltimore, Maryland; University of Pittsburgh, Pittsburgh, PA; VA Pittsburgh Healthcare System, Pittsburgh, Pennsylvania; University of Maryland School of Medicine, Baltimore, Maryland

## Abstract

**Background:**

The risk of MRSA transmission differs by healthcare personnel (HCP) role and care activity. An alternative to an “all or none” approach to contact precautions for patients with MRSA carriage is a “risk-tailored” approach – using gloves and gowns only for certain high-risk activities, locations, or roles. A discrete choice experiment is a proven method to assess HCP perspectives on varied implementation strategies.

**Figure:**
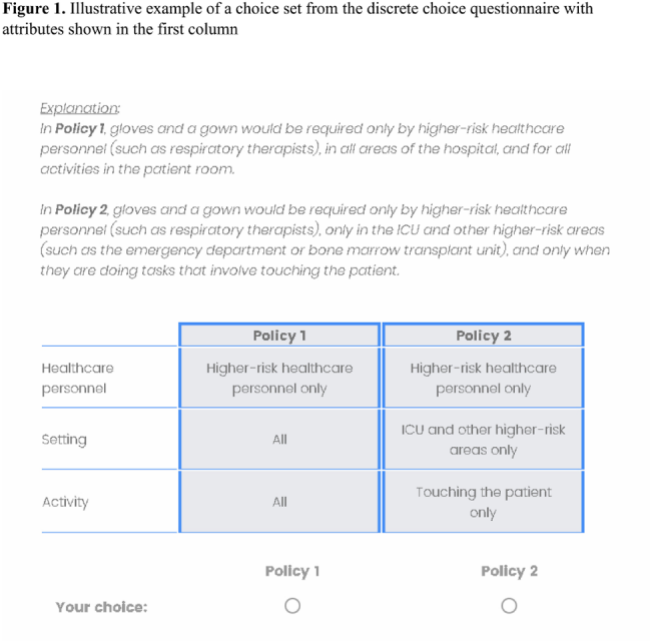

**Methods:**

We distributed a discrete choice experiment survey to HCPs at medical centers in three cities. Respondents were presented with eight choice sets, each consisting of two hypothetical policy options for glove and gown use to prevent MRSA transmission (Fig 1). In each comparison, respondents selected their preferred policy option. Using mixed logit modeling we calculated the utility derived from each policy component, the probability of uptake for the most favored policies, and heterogeneity in preferences based on HCP role.

**Figure:**
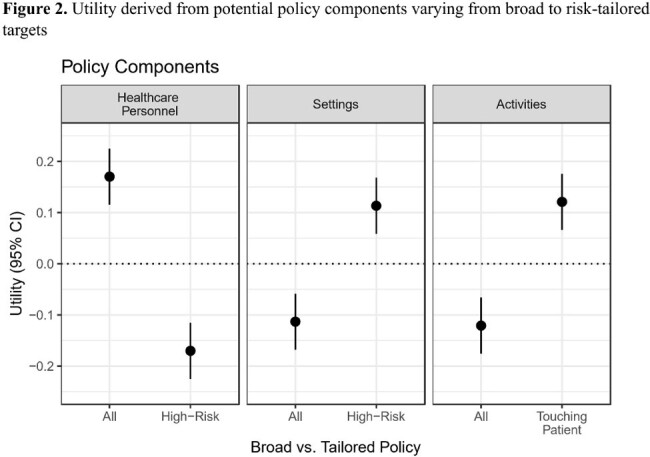

**Results:**

384 HCP completed the survey: 138 from New York, 128 from Pittsburgh, and 118 from Baltimore. Respondents’ occupations were physicians or advanced practice providers (93, 24%), nurses (87, 23%), environmental services (56, 15%), respiratory therapists (55, 14%), occupational or physical therapists (44, 11%), and “other” (46, 11%). 237 (54%) respondents reported wearing gloves and gowns ‘all the time’ when required. Respondents preferred policy with the highest utility score was to use gloves and gown for all HCPs roles (utility, 0.17; 95% CI, 0.12 to 0.23), in high-risk settings (utility, 0.12; 95% CI 0.07-0.18), when touching the patient (utility, 0.11; 95% CI 0.06-0.17) (Fig 2). Sixty-three percent (95% CI 60-66%) of respondents would support a risk-tailored approach over an approach where contact precautions are used by all HCP in all settings and for all activities. The support for this policy varied by HCP role (p< 0.02), with the strongest probability of support from physicians and advanced practice providers (77%, 95% CI 72-82%) and the least support from environmental services (45%, 95% CI 37-53%) (Fig 3).

**Figure:**
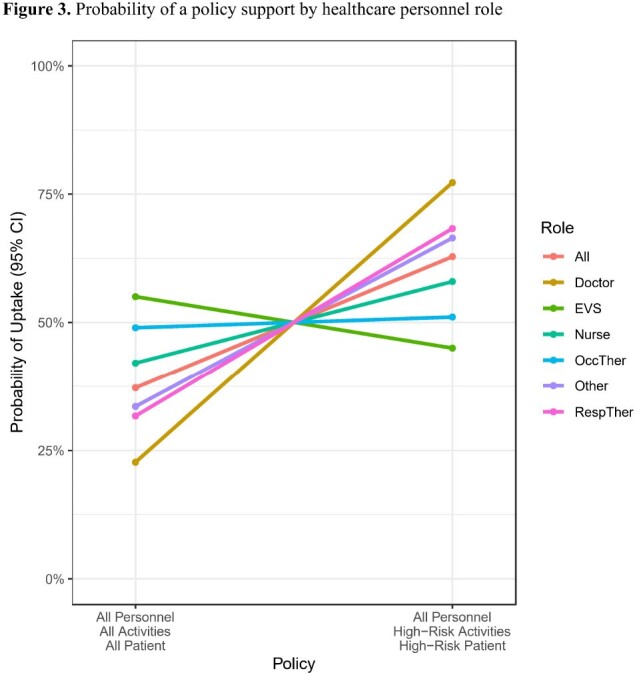

**Conclusion:**

This discrete choice survey demonstrates that most HCP prefer a risk-tailored approach to contact precautions when caring for patients with MRSA.

**Disclosures:**

**Anthony Harris, MD, MPH**, Innoviva: Advisor/Consultant|UpToDate: Infection Control Editor **Graham M. Snyder, MD, SM**, Infectious Diseases Connect: Advisor/Consultant **Nathan N. O'Hara, PhD MHA**, Arbutus Medical Inc.: Stocks/Bonds (Private Company)

